# Effect of Agroecosystems on Seroprevalence of St. Louis Encephalitis and West Nile Viruses in Birds, La Pampa, Argentina, 2017–2019

**DOI:** 10.3201/eid2807.211485

**Published:** 2022-07

**Authors:** Ana P. Mansilla, Juan M. Grande, Adrián Diaz

**Affiliations:** Consejo Nacional de Investigaciones Científicas y Técnicas, Buenos Aires, Argentina (A. P. Mansilla, J. M. Grande, A. Diaz); Universidad Nacional de La Pampa, Santa Rosa, Argentina (A. P. Mansilla, J. M. Grande);; Universidad Nacional de Córdoba, Córdoba, Argentina (A. Diaz)

**Keywords:** St. Louis encephalitis virus, West Nile virus, seroprevalence, avian hosts, land use, anthropogenic changes, agroecosystems, La Pampa, Argentina, viruses, vector-borne infections

## Abstract

In Argentina, the Pampa ecoregion has been almost completely transformed into agroecosystems. To evaluate the environmental (agricultural area, tree coverage, distance to the nearest water body and urban site) and biological (dove, cowbird, and sparrow abundance) effects on free-ranging bird exposure to St. Louis encephalitis virus (SLEV) and West Nile virus (WNV), we used generalized linear mixed models. For 1,019 birds sampled during 2017–2019, neutralizing antibodies were found against SLEV in samples from 60 (5.8%) birds and against WNV for 21 (2.1%). The best variable for explaining SLEV seroprevalence was agricultural area, which had a positive effect; however, for WNV, no model was conclusive. Our results suggest that agroecosystems in the La Pampa ecoregion increase the exposure of avian hosts to SLEV, thus potentially increasing virus activity.

Zoonotic infections, particularly those transmitted from one vertebrate to another by an arthropod vector (vectorborne diseases), have been frequently identified among the most common emerging infectious diseases (*1*). During recent decades, reemergence of many such pathogens (e.g., dengue virus, yellow fever virus, Zika virus, chikungunya virus, Saint Louis encephalitis virus [SLEV], and West Nile virus [WNV]) represents a threat to human health and wildlife conservation (*2*).

SLEV and WNV belong to the family *Flaviviridae*, genus *Flavivirus*. SLEV is endemic to the Americas and has recently re-emerged in the western United States (*3*,*4*), southern Brazil, and central Argentina (*5*). In Argentina, according to ecologic studies, the SLEV transmission network is integrated by *Culex quinquefasciatus*, *Culex interfor*, and *Culex saltanensis* mosquitoes as vectors (*6*) and eared doves (*Zenaida auriculata*) and Picui ground doves (*Columbina picui*) as amplifying urban hosts (*7*). WNV was first detected in the Americas in 1999, causing an encephalitis outbreak among humans and massive mortality events among American crows (*Corvus brachyrhynchos*) (*8*). In 2006 in Argentina, the virus was isolated from sick horses in Buenos Aires and Entre Ríos Provinces (*9*). However, serologic evidence from free-ranging birds indicates previous endemic WNV activity in a large mosaic of resident birds from central and northern Argentina since 2004 (*10*). Vector competence studies have indicated that *Cx. quinquefasciatus* and *Cx. interfor* mosquitoes are able to transmit the local strain of WNV (*11*), whereas host competence studies have identified the Picui ground dove as an amplifier host for WNV (*12*). This finding suggests that ecologic requirements for maintenance could be similar for both viruses.

Land-use changes can affect disease dynamics by modifying the abundance, distribution, behavior, movement, immune response, and community composition of vectors and hosts as well as interactions between vectors and hosts (*13*). In Argentina, the expansion of agriculture into native ecosystems has generated great modifications of the landscape and the biological communities that inhabit these regions. Specifically, because of the aptitude of its soils, the Pampean region, located in the central-eastern part of Argentina, is one of the areas most greatly modified by human activities. This area has almost entirely been converted to large-scale agricultural land, which in turn has generated changes in the abundance of small mammals and birds (*14*). However, some species of rodents and native doves have successfully adapted to these changes and, because of their abundance, are considered agricultural pests (*15*). The large populations of several columbid species, such as eared doves, Picui ground doves, and spot-winged pigeons (*Patagioenas maculosa*), could generate appropriate ecologic conditions for increased SLEV and WNV activity. In this context, our goal was to study the exposure of free-ranging bird communities to SLEV and WNV and to evaluate environmental and biological factors potentially associated with that exposure in agroecosystems in the Pampean region of Argentina.

Bird capture, manipulation, banding, and blood sampling were authorized by the Direction of Natural Resources belonging to the Subsecretary of Agrarian Affairs from the Ministry of Production of La Pampa Province. Birds were handled according to the guidelines for the use of wild birds in research elaborated by the Ornithological Council (https://birdnet.org/wp content/uploads/2017/07/guc3adas-para-la-utilizacion-de-aves-silvestres-en-investigacic3b3n.pdf). Field studies did not involve endangered or protected species.

## Methods

### Study Area and Sampling Sites

**Figure 1:**
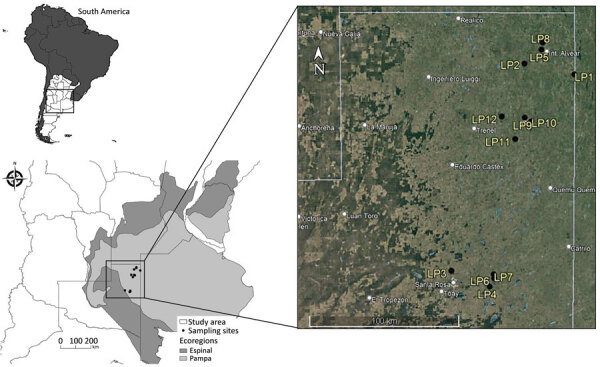
Sampling sites study of effect of agroecosystems on seroprevalence of St. Louis encephalitis and West Nile viruses in birds in the Pampean Grasslands, northeastern La Pampa Province, Argentina. Inset map at top left shows location of sites in South America. LP, La Pampa.

We conducted this study in the northeastern region of La Pampa Province, Argentina, during the period of arbovirus activity (February–April) in 2017–2019. Within the study area, we selected sampling sites for bird captures randomly and included only those with permission from land owners and a minimum distance of 2,000 meters between each other, leading to a total of 12 sampling sites (Figure 1). The study area was formerly part of the Pampean grasslands ecoregion but has been entirely transformed to agriculture. The Pampean grasslands was a vast treeless plain covered by a variety of grasses, such as *Sorghastrum pellitum* and *Elionurus muticus* (*16*). In La Pampa Province, this area is now almost completely transformed, dominated by an agricultural exploitation system based on intensive soybean cultivation via direct sowing methods. Wheat (generally alternated with soybean in the same year), sunflowers, and corn are also cultivated, although to a lesser extent; some plots are seminatural or implanted pastures for cattle (*17*). Toward the center of the province, soybean cultivation is less common and seminatural pastures dominate the landscape, alternating with different crops such as wheat, corn, and sunflowers. This central area also contains some small isolated patches of Caldén (*Prosopis caldenia*) forest in the transition to the Espinal ecoregion (Figure 1). Across the study area, but more markedly in the northeastern region, settlements are surrounded by non-native tree woodlots (sometimes up to 20–30 hectares), which constitute a key element in the presence and abundance of pest birds, such as eared doves (*15*). The climate is dry subhumid; rainfall is distributed throughout the year, but the highest monthly precipitation is in the summer (October–March), increasing in a southwest-to-northeast gradient (*18*).

### Bird Collection and Serum Samples

At each site, we operated 7 mist nets for 3 or 4 days during dawn and late afternoon. We banded collected birds with numbered aluminum leg bands displaying the shipping address of the Argentine Museum of Natural Sciences provided by the Aves Argentinas association (https://www.avesargentinas.org.ar). By using a specialized field guide for bird species from Argentina and Uruguay (*19*), we recorded species, age, sex, and regular morphometric measurements for each bird. We collected blood by jugular (most species) or brachial (columbids) venipuncture, with 27-gauge sterile needles, into plastic tubes containing 0.45 mL or 0.9 mL (according to a sample volume of 0.1 mL or 0.2 mL) of minimum essential medium for a serum dilution of ≈1:10. We held tubes at room temperature for 20–30 min for coagulation and then placed them into coolers. At the laboratory, we centrifuged samples at 5,000 × *g* for 15 min for serum separation and then stored them at −20°C. Before releasing the birds, we hydrated those sampled with sugar water. We did not collect blood from birds weighing <10 g.

### Serologic Assays and Data Interpretation

We analyzed serum samples to detect neutralizing antibodies by using the plaque-reduction neutralization test. We used low-passage strains of SLEV CbaAr-4005 and WNV E/7229/06. The SLEV CbaAr-4005 strain was isolated from *Cx. quinquefasciatus* mosquitoes collected in Córdoba Province (*20*), and the WNV E/7229/06 strain was isolated from a dead horse in Buenos Aires Province, Argentina (*9*).

We considered all serum samples that neutralized >80% of the inoculated plaque-forming units to be positive and subjected samples that were positive for both viruses to titration (*21*). We prepared 7 serial 2-fold dilutions of serum, resulting in final dilutions of 1:20, 1:40, 1:80, 1:160, 1:320, 1:640, and 1:1,280. We assigned endpoint titers as the reciprocal of the greatest dilution in which >80% of the challenge virus was neutralized. According to experiments that evaluated cross-reaction between SLEV and WNV in heterologous inoculation scenarios in common quail (*Coturnix coturnix*), which indicated no cross-reaction between SLEV and WNV (A. Diaz, unpub. data), as well as evidence provided by Patiris et al. (*22*) and Ledermann et al. (*23*), we considered all serum samples with antibody titers >20 to be positive. Therefore, we considered samples with titers >20 for both viruses to indicate multiple heterologous infections.

### Environmental and Biological Data

To determine the influence of different environmental and biological variables on SLEV and WNV seroprevalence, we built a buffer area with a radius of 1.5 km around the sampling sites, within which we calculated the area occupied by various classes of land cover and other variables of interest. We based our buffer of 1.5 km on the dispersal patterns of several mosquitoes of the genus *Culex*, particularly *Cx. quinquefasciatus* (*24*), and some species of territorial birds, such as house sparrows (*Passer domesticus*), rufous-collared sparrows (*Zonotrichia capensis*), and rufous horneros (*Furnarius rufus*). We used SPOT 6 images granted by the National Commission for Space Activities (https://www.argentina.gob/ar/ciencia/conae). On these images we created a shape or layer file on which polygons corresponding to the different classes of land cover were digitized. Within the buffer area, we estimated the area and distances to variables relevant for arbovirus transmission (Table 1). We used the free open software QGIS version 3.4.10 (https://www.qgis.org) for all GIS procedures and analyzed the following environmental variables: agricultural area (which included crops and pasture lands) expressed in square kilometers; tree coverage (which included native forest patches and non-native tree woodlots) expressed in square kilometers; distance to the nearest water body (expressed in kilometers); and distance to the closest urban settlement (expressed in kilometers) (Tables 1, 2). On the basis of previous host competence studies (*7*), we considered as biological variables the abundance of doves (eared doves, Picui ground doves, and spot-winged pigeons), cowbirds (grayish baywings [*Agelaioides badius*] and shiny cowbirds [*Molothrus bonariensis*]), and house sparrows, considering the abundance as the total number of individuals of each species counted on each sampling site. Dove, cowbird, and sparrow abundance was estimated according to observational and acoustic bird counts on each site, for which we used the fixed width transect method of 50 × 200 m and performed 6 transects in each site according to a rarefaction analysis. The 6 transects were randomly distributed to cover as much of the site as possible and were >200 m apart to minimize possible biases by double counting of birds (*25*). All linear transects were surveyed once by the same single observer. Bird surveys took place during March and April 2018 and 2019, from 6:00 to 10:00 a.m.

**Table 1:** Models proposed to analyze the association between environmental and biological variables and SLEV/WNV seroprevalence in birds*

Model no.†	Variables	Biological justification
1/9	Null model	The environmental and biological variables considered in this study do not explain the SLEV/WNV seroprevalence.
2/10	Distance to water body	The water bodies are favorable habitats for the development of immature mosquitoes, especially of the genus *Culex*, for which a greater abundance of potential mosquito vectors will be generated in these sites. Also, birds use these sites for drinking, facilitating the encounter between hosts and vectors.
3/11	Agricultural area	Places with a homogeneous agricultural matrix will have impoverished biological communities dominated by birds of a few species, such as eared doves (*Zenaida auriculata*) and Picui ground doves (*Columbina picui*) with the potential to amplify viruses.
4/12	Distance to urban site	Peri-urban areas present better conditions for the establishment of different *Culex* mosquito species, generating a greater abundance of potential vectors.
5/13	Dove abundance	Host competence assays identified columbiform species as the main amplifying hosts for SLEV and WNV in Argentina, so a greater abundance of these species will produce greater virus circulation in those sites.
6/14	Sparrow abundance	House sparrow populations in Córdoba Province were not very efficient at amplifying SLEV, so a higher abundance of birds of this species would generate a viral dilution effect at the sites.
7/15	Agricultural area + dove abundance	Doves have a high capacity to amplify SLEV and WNV and are very abundant in disturbed environments occupied by crops and pastures, providing greater virus circulation in those places.
8/16	Distance to water body + agricultural area	Places that have larger agricultural areas and are closer to water bodies will have impoverished biological communities dominated by eared doves and Picui ground doves and high mosquito abundance.

*SLEV, St. Louis encephalitis virus; WNV, West Nile virus. †Models 1–8 correspond to those proposed for SLEV and 9–16 correspond to those proposed for WNV.

**Table 2:** Number of positive/total SLEV/WNV samples collected per site, seroprevalence in birds, and environmental and biological variables in study of effect of agroecosystems on seroprevalence of SLEV and WNV in birds, La Pampa, Argentina, 2017–2019*

Site	SLEV	%, (95% CI)	WNV	% (95% CI)	TC, km^2^	AA, km^2,^	UD, km	WD, km	DA†	SA‡	CA§
LP1	18/63	28.57 (17.8–41.3)	4/63	6.35 (1.7–15.4)	0.17	6.89	8.95	2.13	56	0	15
LP2	17/100	17 (10.2–25.8)	6/100	6 (2.2–12.6)	0.22	6.83	2.04	2.06	109	50	40
LP3	6/78	7.69 (2.8–15.9)	2/78	2.56 (0.3–8.9)	0.51	6.55	3.25	7.17	115	0	18
LP4	5/107	4.67 (1.5–10.5)	0/107	0 (0–3.4)	0.89	6.17	12.98	3.22	196	0	46
LP5	0/104	0 (0–3.5)	0/104	0 (0–3.5)	0.11	6.95	2.45	0.59	22	120	150
LP6	0/101	0 (0–3.5)	1/101	0.99 (0.02–5.4)	0.47	6.59	7.95	6.31	926	53	520
LP7	1/85	1.17 (0.6.3)	2/85	2.35 (0.2–8.2)	0.36	6.7	5.28	3.45	186	0	255
LP8	0/71	0 (0–5)	0/71	0 (0–5)	0.04	7.02	5.2	0.62	37	5	30
LP9	1/73	1.37 (0.03–7.3)	0/73	0 (0–4.9)	0.48	6.57	0.36	1.34	120	50	27
LP10	1/89	1.12 (0.02–6.1)	0/89	0 (0–4)	0.1	6.96	1.16	0.83	73	20	32
LP11	4/60	6.66 (1.8–16.1)	3/60	5 (1–13.9)	0.03	7.03	9.3	0.05	82	40	27
LP12	7/88	7.95 (3.2–15.7)	3/88	3.41 (0.7–9.6)	0.004	7.06	12.56	0.32	47	30	37

*AA, agricultural area; CA, cowbird abundance; DA, dove abundance; SA, sparrow abundance; SLEV, St. Louis encephalitis virus; TC, tree coverage; UD, distance to the closest urban settlement; WD, distance to the nearest water body; WNV, West Nile virus, †No. eared doves (*Zenaida auriculata*) + Picui ground doves (*Columbina picui*) + spot-winged pigeons (*Patagioenas maculosa*). ‡No. house sparrows (*Passer domesticus*). §No. grayish baywings (*Agelaioides badius*) + shiny cowbirds (*Molothrus bonariensis*).

### Statistical Analyses

We estimated SLEV and WNV activities by means of neutralizing antibody prevalence. We calculated seroprevalence and 95% CIs by using the package binom (*26*) and the Pearson-Klopper method within R software (https://www.r-project.org). We analyzed associations between sampling sites, bird species, and exposure to SLEV/WNV through generalized linear mixed models (GLMM) with binomial distribution, in which the sampling year was considered as a random factor. We compared seroprevalence values for each virus evaluated in seropositive birds of each species by using the Pearson χ^2^ test. We considered p values to be significant at a threshold of α = 0.05. We investigated the association between environmental and biological variables and the SLEV/WNV seroprevalence at each sampling site by using GLMM with binomial error distribution and logit link function, considering the sampling year as a random variable in all models. We evaluated collinearity between explanatory variables by using Pearson correlation with r >0.60 as a limit (Table 1). Because the environmental variables “agricultural area” and “tree coverage” were strongly correlated (r = −0.99), we removed the second variable from the set of models proposed, and because we found the same correlation for the variables “dove abundance” and “cowbird abundance” (r = 0.85), we eliminated “cowbird abundance” from the analyses. The model was selected by using the Akaike information criterion (AIC) and its corrected calculation for small sample sizes (AICc) (*27*). We compared models by using ΔAICc, which is the difference between the lowest AICc value (as the best of suitable models) and the AICc from all other models. Competing models were those differing by ΔAICc ≤2 from the top model, and Akaike weights (*w*) were an indication of support for each model. We evaluated the support for the performance of individual predictor variables by summing the AICc weight of a model (*w*_i_) across all models that contained the parameter being considered (*27*). To evaluate the support for parameter estimates, we calculated 95% CIs by using unconditional variances and assumed the considered variable assumed to be significantly associated with the SLEV/WNV seroprevalence when the 95% CI excluded zero (*27*).

## Results

**Figure 2:**
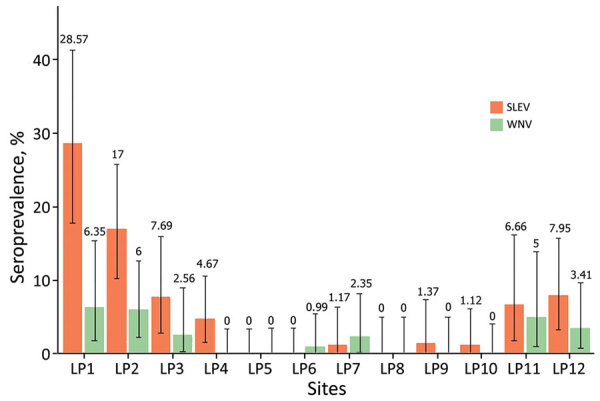
Spatial distribution of the seroprevalence of neutralizing antibodies for St. Louis encephalitis virus (SLEV) and West Nile (WNV) virus in free-ranging birds collected in 12 sampling sites in La Pampa province, Argentina (see Figure 1). Numbers above bars indicate specific seroprevalence for each site; error bars indicate 95% CIs. LP, La Pampa; SLEV, Saint Louis encephalitis virus; WNV, West Nile virus.

Of the 1,019 free-ranging birds belonging to 44 species collected and sampled, seroprevalence rates were 5.8% (60/1,019) for SLEV and 2.1% (21/1,019) for WNV. Neutralizing antibody titers were >20 for both viruses for 12 birds, which were thus considered to have multiple heterologous infections. Of the 12 sites sampled, birds were seropositive for SLEV at 9 sites, for WNV at 7, and for both viruses at 6 (Table 2, Figure 2).

The GLMM performed to analyze the associations between the sampling sites, avian species, and exposure to SLEV/WNV, showed that sampling site was a significant variable affecting seroprevalence of SLEV (p = 5.87 × 10^–16^ and WNV (p = 0.0012); seroprevalence for both viruses was highest at sites in the northern area (Figures 1, 2). However, bird species did not significantly influence seroprevalence of SLEV (p = 0.50) or WNV (p = 0.72).

Birds of 17 species were seropositive for SLEV and of 8 species for WNV. Species most exposed to SLEV were house wrens (*Troglodytes aedon*), chalk-browed mockingbirds (*Mimus saturninus*), monk parakeets (*Myiopsitta monachus*), eared doves, and house sparrows (Table 3), whereas those most exposed to WNV were monk parakeets, rufous horneros, and grayish baywings. We found no significant statistical difference between the viruses among seropositive birds of different species, except for house sparrows (p = 0.0004).

**Table 3:** SLEV/WNV species-specific seroprevalence in birds collected in different agroecosystems in study of effect of agroecosystems on seroprevalence of SLEV and WNV in birds, La Pampa Province, Argentina, 2017–2019*

Species	SLEV			WNV	
	No. pos./no. tested	% Positive (95% CI)†		No. pos./no. tested	% Positive (95% CI)†
House sparrow (*Passer domesticus*)	17/237	7.17 (4.23–11.23)		2/237	0.84 (0.10–3.01)
Rufous-collared sparrow (*Zonotrichia capensis*)	9/181	4.97 (2.29–9.23)		0/181	0 (0–2.02)
Rufous hornero (*Furnarius rufus*)	6/93	6.45 (2.40–13.51)		7/930	7.52 (3.08–14.89)
Picui ground dove (*Columbina picui*)	5/100	5 (1.64–11.28)		4/100	4 (1.10–9.92)
Grayish baywing (*Agelaioides badius*)	2/63	3.17 (0.38–11)		3/63	4.76 (0.99–13.29)
Monk parakeet (*Myiopsitta monachus*)	2/25	8 (0.98–26.03)		2/25	8 (0.98–26.03)
Eared dove (*Zenaida auriculata*)	2/26	7.69 (0.94–25.13)		1/26	3.84 (0.09–19.63)
Shiny cowbird (*Molothrus bonariensis*)	0/72	0 (0–4.99)		1/72	1.38 (0.03–7.49)
House wren (*Troglodytes aedon*)	6/63	9.52 (3.57–19.58)		0/63	0 (0–5.68)
Double-collared seedeater (*Sporophila caerulescens*)	1/19	5.26 (0.13–26.02)		0/19	0 (0–17.64)
Grassland yellow finch (*Sicalis luteola*)	1/20	5 (0.12–24.87)		0/20	0 (0–16.84)
Chalk-browed mockingbird (*Mimus saturninus*)	1/12	8.33 (0.21–38.47)		0/12	0 (0–26.46)
Tropical kingbird (*Tyrannus melancholicus*)‡	2/4	–		0/4	–
American kestrel (*Falco sparverius*)‡	2/4	–		0/4	–
Saffron finch (*Sicalis flaveola*)‡	1/6	–		0/6	–
Pale-breasted spinetail (*Synallaxis albescens*)‡	1/1	–		0/1	–
White-winged black tyrant (*Knipolegus aterrimus*)‡	1/1	–		1/1	–
Hudson´s black tyrant (*Knipolegus hudsoni*)‡	1/1	–		0/1	–
Grassland sparrow (*Ammodramus humeralis*)	0/5	–		0/5	–
Firewood-gatherer (*Anumbius annumbi*)	0/4	–		0/4	–
Sharp-billed canastero (*Asthenes pyrrholeuca*)	0/1	–		0/1	–
Hooded siskin (*Spinus magellanicus*)	0/1	–		0/1	–
Buff-winged cinclodes (*Cinclodes fuscus*)	0/1	–		0/1	–
Dark-billed cuckoo (*Coccyzus melacoryphus*)	0/3	–		0/3	–
Green-barred woodpecker (*Colaptes melanochloros*)	0/4	–		0/4	–
Rufous-browed peppershrike (*Cyclarhis gujanensis*)	0/1	–		0/1	–
White-crested elaenia (*Elaenia albiceps*)	0/1	–		0/1	–
Guira cuckoo (*Guira guira*)	0/1	–		0/1	–
Narrow-billed woodcreeper (*Lepidocolaptes angustirostris*)	0/3	–		0/3	–
Cattle tyrant (*Machetornis rixosa*)	0/4	–		0/4	–
Patagonian mockingbird (*Mimus patagonicus*)	0/1	–		0/1	–
White-banded mockingbird (*Mimus triurus*)	0/1	–		0/1	–
Screaming cowbird (*Molothrus rufoaxillaris*)	0/12	–		0/12	–
Spot-winged pigeon (*Patagioenas maculosa*)	0/2	–		0/2	–
Great kiskadee (*Pitangus sulphuratus*)	0/27	–		0/27	–
Brown cacholote (*Pseudoseisura lophotes*)	0/1	–		0/1	–
Vermilion flycatcher (*Pyrocephalus rubinus*)	0/8	–		0/8	–
Roadside hawk (*Rupornis magnirostris*)	0/1	–		0/1	–
Golden-billed saltator (*Saltator aurantiirostris*)	0/1	–		0/1	–
Greater wagtail-tyrant (*Stigmatura budytoides*)	0/1	–		0/1	–
Southern scrub-flycatcher (*Sublegatus modestus*)	0/3	–		0/3	–
Sooty-fronted spinetail (*Synallaxis frontalis*)	0/2	–		0/2	–
Chaco earthcreeper (*Tarphonomus certhioides*)	0/1	–		0/1	–
Blue-crowned parakeet (*Thectocercus acuticaudatus*)	0/1	–		0/1	–

*Dashes indicate that seroprevalence was not estimated because of the small number of serum samples tested for birds of that species. SLEV, St. Louis encephalitis virus; WNV, West Nile virus. †Prevalence (%) defined as the no. positives divided by total samples multiplied by 100. ‡For seropositive species with <10 birds, seroprevalence was not calculated.

The best model explaining the variation in SLEV seroprevalence included the agricultural area as an explanatory variable (*w*_i_ = 0.44; Table 4). SLEV seroprevalence increased with agricultural area (Table 5). Odds ratio for this model was 1.97, which means that for each unit of increase in agricultural area size, SLEV seroprevalence increased an average of 1.97 times. The model that best explained the variation in WNV seroprevalence included the distance to the nearest water body and agricultural area as explanatory variables (*w*_i_ = 0.37; Table 6), but neither of the 2 variables was statistically significant to explain the variation in WNV seroprevalence because both 95% CIs included zero (Table 7).

**Table 4:** Models for SLEV seroprevalence based on the generated hypotheses ranked by their AIC scores in study of effect of agroecosystems on seroprevalence of SLEV and WNV in birds, La Pampa, Argentina, 2017–2019*

Model	Variables of the model	*k*	AICc	∆AICc	*w_i_*
GLMM3	Agricultural area	3	388.819	0.000	0.441
GLMM8	Distance to water body + agricultural area	4	390.198	1.379	0.221
GLMM7	Agricultural area + dove abundance	4	390.244	1.425	0.216
GLMM5	Dove abundance	3	391.421	2.602	0.120
GLMM2	Distance to water body	3	401.845	13.026	0.001
GLMM1	Null model	2	408.710	19.892	0.000
GLMM6	Sparrow abundance	3	410.216	21.397	0.000
GLMM4	Distance to urban site	3	410.465	21.647	0.000

*AICc, Akaike information criterion corrected for small samples; ∆AICc, difference between AICc and the AICc from all other models; *k*, number of estimated parameters; SLEV, St. Louis encephalitis virus; *w_i_***,** relative likelihood that the specific model is the best of the suite of all models; WNV, West Nile virus.

**Table 5:** Parameter data for explanatory variables describing variation in SLEV seroprevalence with ∆AICc <2 in study of effect of agroecosystems on seroprevalence of SLEV and WNV in birds, La Pampa, Argentina, 2017–2019*

Explanatory variable	Parameter likelihood	Parameter estimate ± SE	95% CI
Intercept		**–3.79 ± 1.10**	**–5.96 to 1.61**
**Agricultural area**	**1.00**	**0.68 ± 0.22**	**0.23 to 1.13**
Distance to water body	0.25	−0.18 ± 0.22	−0.63 to 0.27
Dove abundance	0.25	−0.87 ± 1.43	−3.68 to 1.94

*Boldface indicates explanatory variables with 95% CIs excluding zero. ∆AICc, difference between the lowest Akaike information criterion corrected for small samples (AICc) and the AICc from all other models; SLEV, St. Louis encephalitis virus; WNV, West Nile virus..

**Table 6:** Models for WNV seroprevalence based on the generated hypotheses ranked by their Akaike information criterion scores in study of effect of agroecosystems on seroprevalence of SLEV and WNV in birds, La Pampa, Argentina, 2017–2019*

Model	Variables of the model	*k*	AICc	∆AICc	*w_i_*
GLMM16	Distance to water body + agricultural area	4	202.760	0.000	0.3762
GLMM11	Agricultural area	3	202.903	0.143	0.34637
GLMM15	Agricultural area + dove abundance	4	204.134	1.374	0.1872
GLMM9	Null model	2	207.459	4.699	0.035
GLMM13	Dove abundance	3	209.011	6.252	0.0156
GLMM10	Distance to water body	3	209.227	6.468	0.014
GLMM12	Distance to urban site	3	209.237	6.478	0.0154
GLMM14	Sparrow abundance	3	209.367	6.607	0.0143

*∆AICc, difference between the lowest Akaike information criterion corrected for small samples (AICc) and the AICc from all other models; SLEV, St. Louis encephalitis virus; WNV, West Nile virus.

**Table 7:** Parameter data for explanatory variables describing variation in WNV seroprevalence with ∆AICc <2 in study of effect of agroecosystems on seroprevalence of SLEV and WNV in birds, La Pampa, Argentina, 2017–2019*

Explanatory variable	Parameter likelihood	Parameter estimate ± SE	95% CI
Intercept		**−4.23 ± 0.53**	**−5.28 to −3.17**
Agricultural area	1.00	1.07 ± 0.61	−0.19 to 2.34
Distance to water body	0.41	0.65 ± 0.50	−0.32 to 1.64
Dove abundance	0.21	0.35 ± 0.36	−0.36 to1.07

*Boldface indicates explanatory variables with 95% CIs excluding zero. ∆AICc, difference between the lowest Akaike information criterion corrected for small samples (AICc) and the AICc from all other models. SLEV, St. Louis encephalitis virus; WNV, West Nile virus.

## Discussion

Our estimations of 6% SLEV and 2% WNV seroprevalence in avian hosts in agroecosystems of La Pampa Province are similar to those detected in and around Córdoba city, Argentina (SLEV 7.73%; WNV 1.47%) (*21*). Composition of biological communities in Córdoba are similar to those in this study.

The species of birds that were infected in the agroecosystems differed according to viruses studied and differed from those found infected by other research conducted in Argentina (*21*,*28*). In our study, the species of birds most infected with SLEV belonged to the families Troglodytidae (house wrens), Mimidae (chalk-browed mockingbirds), and Passeridae (house sparrows), although in other studies of similar characteristics and conducted in temperate and subtropical regions of Argentina, the species most infected with this virus belonged to the families Columbidae, Furnariidae, Icteridae, and Tyrannidae (*21*,*28*). For WNV, the most infected birds in our study were rufous horneros, which had already been highlighted as maintenance hosts for WNV in central Argentina (*21*), and monk parakeets, for which WNV infection had not been detected in other studies. One of the main amplifying hosts for SLEV in the United States and for WNV in Europe is the house sparrow (*29*,*30*). In previous studies conducted in the northeastern region of Argentina, SLEV seropositivity was not detected in >200 serum samples collected from house sparrows (*28*). Moreover, in urbanized temperate areas of the central region of Argentina, such as Córdoba, seroprevalence rates for house sparrows have been low for both viruses (3.92% for SLEV, 1.96% for WNV) (*21*). Furthermore, although the host competence index value for house sparrows is low (*7*), their high abundance and high exposure to SLEV observed in our study would indicate an efficient role as amplifying hosts for SLEV in agricultural areas of La Pampa Province. A possible explanation for the differences observed among the exposed bird species of and between disturbed environments (agricultural and urban) could be the presence of different vector mosquito species for the viruses evaluated with different host-feeding preferences. Changes in land use could also modify the host-seeking behavior of mosquitoes affecting avian host exposure to vectored viruses (*31*).

Although seroprevalence values in our study were low, seropositive bird species are resident and seropositive birds were detected during the 3 years sampled. This finding probably indicates endemic circulation for both viruses in this region of Argentina. Previous study of SLEV and WNV activities recorded in Pampean agricultural systems also showed low levels of exposure but in a particular group of birds, the birds of prey (*32*).

In our study, SLEV seroprevalence was positively associated with the agricultural area, and thus, inversely correlated by tree cover. In other studies, contrary to our results, SLEV infection in humans has been positively associated with proximity to areas with highly productive vegetation cover estimated by the Normalized Difference Vegetation Index (*33*,*34*), low density urban construction, and the distance to agricultural fields (*34*). These differences could be explained because the variables of interest differ (SLEV infections in humans vs. seroprevalence among birds) and the explanatory variables were also considered differently. In our study, we considered the area occupied by agricultural activities and tree coverage within a buffer of interest; in the other studies, researchers considered the distances between cases of SLEV infection in humans and the environmental variables. In turn, the differences found could also result from the fact that the tree coverage in our study area is mostly characterized by planted nonnative tree woodlots, which are inherently different from the green spaces or patches of native forest that characterized the vegetation in other studies.

The model that best explained the variation in WNV seroprevalence included the distance to the nearest water body and agricultural area as explanatory variables, but neither of the 2 variables explained the variation in WNV seroprevalence with statistical significance. Land use effect on WNV activity has been extensively studied, at least in the United States (*35*–*41*). Studies have shown that the abundance and distribution patterns of the mosquito vector are key factors in determining virus activity; and these, in turn, are greatly affected by land use. For example, in the northeastern United States, where the main vectors are *Cx. pipiens* and *Cx. quinquefasciatus* mosquitoes, urbanization positively affects the incidence of WNV disease in humans (*35*), whereas on the west coast of the United States, where the most efficient vectors are *Cx. tarsalis* mosquitoes, the main land cover types associated with increased WNV activity are agricultural irrigated areas, such as rice fields and orchards (*40*).

Anthropogenic activities are among the most influential factors affecting emergence of infectious diseases, particularly viral vectorborne zoonoses. Viruses carried by *Aedes* mosquitoes (e.g., chikungunya, dengue, and Zika viruses) are positively affected by urbanization as the main breeding substrates of *Ae. aegypti* and *Ae. albopictus* mosquito vectors, which become highly abundant in those anthropogenic and urban habits (*42*,*43*). However, for viruses carried by *Culex* mosquitoes (e.g., Japanese encephalitis virus, WNV, SLEV, and Usutu virus), how anthropogenic changes affect virus activity is not well known. The generalist host-feeding and host-seeking behavior and wide tolerance for rearing sites of the *Culex* mosquito vectors make it difficult to determine the effect of land use on the activity of *Culex* mosquitoborne viruses.

Our findings suggest that modified ecosystems, such as agroecosystems in La Pampa Province, have the environmental and biological factors necessary for maintaining and amplifying re-emerging viruses such as SLEV and WNV. However, our study did not analyze the change in land use but rather focused on how the current elements of the already modified landscape influence biological communities and, consequently, SLEV and WNV activity. The sites considered in this study were limited, and the environmental characterization was conducted extensively without taking into account, for example, the identity of the crops or pastures within the agricultural areas. Furthermore, because the seroprevalence data for birds do not necessarily reflect the place or the time in which they were infected, this information should be used with caution and complemented with studies on viral activity in the mosquito communities that ensures circulation of the virus at a certain time and place. Although further research on the ecology and biology of these viruses is needed to determine how crop production, monoculture areas, and associated landscapes affect vector transmission dynamics of these viruses, we conclude that the Pampean agroecosystems in Argentina affect SLEV seroprevalence among avian hosts, providing evidence of the effect of land use on the activity of arboviruses.
